# Perceived Racial Discrimination and Marijuana Use a Decade Later; Gender Differences Among Black Youth

**DOI:** 10.3389/fped.2019.00078

**Published:** 2019-03-22

**Authors:** Shervin Assari, Ritesh Mistry, Daniel B. Lee, Cleopatra Howard Caldwell, Marc A. Zimmerman

**Affiliations:** ^1^Department of Family Medicine, Charles R. Drew University of Medicine and Science, Los Angeles, CA, United States; ^2^Department of Psychiatry, University of Michigan, Ann Arbor, MI, United States; ^3^Center for Research on Ethnicity, Culture and Health, School of Public Health, University of Michigan, Ann Arbor, MI, United States; ^4^Department of Health Behavior and Health Education, School of Public Health, University of Michigan, Ann Arbor, MI, United States; ^5^Prevention Research Center, School of Public Health, University of Michigan, Ann Arbor, MI, United States

**Keywords:** blacks, African Americans, gender, racial discrimination, substance use

## Abstract

**Background:** Researchers have reported gender differences in the association between perceived racial discrimination (PRD) and substance use including marijuana use (MU). A limited number of longitudinal studies, however, have documented the long-term effect of PRD during adolescence on subsequent MU in young adulthood.

**Objective:** In the current longitudinal study, we tested gender differences in the association between baseline PRD during adolescence and subsequent MU during young adulthood within Black population.

**Methods:** A cohort of 595 Black (278 male and 317 female) ninth grade students were followed for 13 years from 1999 (mean age 20) to 2012 (mean age 33). Participants were selected from an economically disadvantaged urban area in the Midwest, United States. The independent variable was PRD measured in 1999. The outcome was average MU between 2000 and 2012 (based on eight measurements). Covariates included age, socio-demographics (family structure, and parental employment), and substance use by friends and parents. Gender was the focal moderator. Linear regression was used for statistical analysis.

**Results:** In the pooled sample, PRD in 1999 was not associated with average MU between 2000 and 2012. We did, however, find an interaction effect between baseline PRD and gender on average MU, suggesting stronger association for males than females. In gender-specific models, baseline PRD predicted average MU between 2000 and 2012 for males, but not for females.

**Conclusion:** Exposure to PRD during late adolescence may have a larger role on MU of male than female Black young adults. Although we found that males are more vulnerable to the effects of PRD on MU, PRD should be prevented regardless of race, gender, and other social identities. While PRD is pervasive among Black Americans, exposure to PRD increase the risk of MU for Black males. Hence, substance use prevention efforts for Black males, in particular, should emphasize coping with PRD.

## Introduction

Perceived racial discrimination (PRD), defined as perceiving unfair and unfavorable treatment due to race, is a chronic stressor that negatively impacts physical (1–6) and mental (7–12) health. Blacks frequently experience PRD over their life course (5, 11, 13–19). PRD adversely affects mental health outcomes (20–22). For instance, researchers have consistently documented the deleterious effect of PRD on state and trait negative affect (23), perceived distress (24), psychiatric disorders (21, 25), depression (26), anxiety (27), suicide (28), pain (29), school performance (30), and substance use (31–33). For instance, after adjusting for demographic and socioeconomic factors, Blacks who experienced PRD across life domains (i.e., at school, getting a job, at work, getting housing, getting medical care, street /public setting, and police/courts) were at a 3 times higher risk of having used marijuana 100 or more times in their lifetime, compared to Blacks who did not report PRD (34).

The harmful effects of PRD, however, may vary by gender, with males being more vulnerable than females to the effects of PRD on depression and substance use (26, 31, 35–38) and females being more sensitive to the effects of PRD on other health behaviors, such as obesity and eating disorders (39). Some scholars have documented gender differences in experience and response to discriminatory events. While Black males are more likely to be discriminated against on the street [for example get stopped by the police], Black females may be more commonly discriminated against in the workplace (40, 41). In line with gender differences in coping in response to stress (42), Black males and females may differ in their likelihood to use substances to cope racism-related stress (43). Among Caribbean Black adolescents, for example, PRD predicted substance use for males, but not females (44). Similar results were found among an ethnically diverse youth cohort/group/community etc. in Maryland (31). Likewise, in another study, PRD exposure was associated with an increased risk of smoking among Black males, but not females (45). Cooper et al. (46) also found that males may have a higher tendency to use substance to cope with PRD. In fact, scholars have discussed some gender-related variations in coping behavior. For example, females report higher levels of internalizing symptoms in the context of stress (including PRD), whereas males report higher levels of externalizing symptoms (e.g., MU use) (46).

Using a longitudinal design, we investigated gender differences in the long term association between baseline PRD during adolescence (In 1999) and average MU (2000–2012) spanning emerging adulthood and adulthood among Black Americans. In line with previous studies that have documented gender differences in exposure (47) and vulnerability (31, 35–37, 48) to PRD, we expected a stronger association between PRD during adolescence and MU during emerging adulthood for male than female Black youth. As most studies have used cross-sectional data or short follow up period, the unique contribution of this study is to generate longitudinal evidence with 18 years of follow up.

## Methods

### Design and Setting

Flint Adolescent Study (FAS) is an 18 years, interview-based prospective cohort study of urban youth in Flint, Michigan. The FAS followed youth at risk of school dropout and substance use during their transition from adolescence to young adulthood. Although detailed methodological information is available elsewhere (49), we summarize the design and setting, participants and sampling, eligibility criteria, interviews, data collection, and follow up here.

### Participants

The sample was composed of youth at risk of school drop-out. Most participants were from working-class families and only 25% of families were composed of biological parents who were married.

### Sampling

Participating youth were selected from the only four main public high schools in Flint (MI) Community Schools. The only public high schools not included were very small schools for youth with special needs.

A non-probability strategy was used to recruit the sample. Participants were enrolled in the fall semester of ninth grade (average age 15), thus all participants were ninth graders at the time of enrolment to the study.

### Eligibility Criteria

Participants were eligible if they had a school-reported grade point average (GPA) of 3.0 or less in their eighth grade. Any history of developmental disability or emotional impairment as diagnosed by the schools were considered as exclusion criteria.

### Analytical Sample in the Current Study

Although the original study included Black (80%), White (17%), and other races (3%) youth, we included only Black youth, as our study is focused on PRD. We excluded individuals who self-identified as White, mixed race, and other races (*n* = 169 or 20%). To minimize the effect of attrition due to the study design involving a long term follow up period, we calculated average MU as our outcome, regardless of duration of the follow up. This strategy was taken to minimize selection bias due to selective attrition in long term cohort studies (50). In the current study, we used data from Wave 1 (year 1994), Wave 4 (year 1997), and Waves 5 to 12 (years 1999–2012). The analytical sample was 595 Black youth (278 males [46.7%] and 317 females [53.3%]) who had data on demographics, SES, PRD, and at least one data point for MU between 1999 and 2012.

### Interviews

All interviews between 1994 and 1997 were face-to-face interviews conducted mostly at schools. Interviews between 2003 and 2008 were either face to face conducted at respondents' homes or community setting or conducted via telephone. Interviews were conducted by trained interviewers who were selected from the community as well as college students. Previous analyses on a broad range of variables have shown no effects of interviewers' race and gender (49). The study had a low refusal rate (*n* = 60) in Year 1. The participants represented 92% of eligible ninth graders who were in Flint public high schools. Interviews lasted about 1 h on average. Demographics and SES were collected during structured interviews. PRD and substance use (MU) measures were collected using paper-and-pencil questionnaires, applied at the end of the interview.

### Follow Up

All participants were followed over time whether they remained in school, moved to another school, or dropped out of school. Participants were interviewed from 1994 to 1997 (average ages 14–17 years old), 1999–2003 (average ages 19–22 years old), and from 2008 to 2012 (average ages 27–29 years old). The age range for youth over the 12 waves of data collection (from 1994 to 2012) was 14–30 years old.

Participants were interviewed annually from 1994 to 1997, 1999–2003, and from 2008 to 2012. The age range for participating youth was 14 years at the start and 32 years at the endpoint of the study. Overall, the study included 12 waves of data collection from 1994 to 2012. Retention rates were 90, 75, and 60% from Waves 1 to 4; Waves 4 to 8, and Waves 9 to 12, respectively.

### Measures

#### Perceived Racial Discrimination (PRD)

We used the Daily Life-Experiences Scale (DLES; 51) to measure PRD in the year 1999. This is a 20-item measure that asks respondents to report if they had experienced racism-related life events or micro-stressors in the past year (51). Some example items include: “Being ignored, overlooked, or not given service (in a restaurant, store, etc.),” “Your ideas or opinions being minimized, ignored, or devalued,” and “Not being hired for a job.” PRD total score was calculated as the mean of all 20 items of the DLES measure, with a potential range from 0 to 5. A higher score was reflective of higher PRD. Cronbach alpha for this scale was 0.94.

#### Marijuana Use (MU)

Participants were asked about the frequency of their marijuana use over the past 30 days at all waves (52). This measure was used between 2000 and 2012. The frequency was rated using a seven-point frequency scale: 1 (none), 2 (1–2 times), 3 (3–5 times), 4 (6–9 times), 5 (10–19 times), 6 (20–39 times), and 7 (more than 40 times). The MU item was drawn from the Monitoring the Future study (53). This variable has been used as outcome in previous research (54–56).

#### Parent Substance Use

A 9-item measure was used to measure Parent substance use. Items included (1) Parents/guardian smoked Marijuana this past year, (2) Parents/guardian get high on drugs, (3) Parents/guardian taken tranquilizers this past year, (4) Parents/guardians taken uppers this past year, (5) Parents/guardian busted for driving high, (6) Parents/guardian busted for using/having drugs, (7) Parents/guardian used heroin, (8) Parents/guardian used cocaine, and (9) Parents/guardian treated for a drug problem. Cronbach alpha for this scale was 0.56. The measure was treated as a dichotomous variable (non-use = 0 vs. use = 1), with 1 reflecting any positive response to the above items, and 0 reflecting a no answer to all items.

#### Friends' Substance Use

A 10-item measure was used to measure friends' substance use. Items included (1) How many of your friends: drug or alcohol problem, (2) How many of your friends: smoke marijuana at least once a month, (3) How many of your friends: have used cocaine, (4) How many of your friends: take pills to get high at least once a month? (5) How many of your friends: use heroin or morphine, (6) How many of your friends: use PCP at least once a month, (7) How many of your friends: sniff glue, paint, or gas at least once a month? (8) How many of your friends: used drugs at school, (9) How many of your friends: busted for selling drugs, and (10) How many of your friends: busted for having drugs. Cronbach alpha for this scale was 0.74. The measure was treated as a dichotomous variable (non-use = 0 vs. use = 1), with 1 reflecting any positive response to the above items, and 0 reflecting a no answer to all items.

#### Sociodemographic Factors

Baseline age, family structure (i.e., non-married parents vs. married parents) and family socio-economic status (number of parents who were employed) were all measured at Wave 1 (year 1994). Age was treated as a continuous measure. SES indicators were dichotomous variables.

### Data Analysis

We used SPSS 20.0 (IBM Corp Armonk, NY) for data analysis. For descriptive purposes, we reported frequency tables (%), as well as mean and standard deviations (SD). Pearson's correlation test was used to estimate bivariate associations between the study variables. For multivariable analysis, we used linear regression models. From our regression models, we reported unstandardized regression coefficients (b), their 95% Confidence Intervals (CI), and associated *p*-values. *p*-values smaller than 0.05 were considered as statistically significant. We used average MU as our outcome, independent of number of observations. So, participants were included in the analysis if they were Black and have reported PRD and at least one observation of MU. In our linear regression models, PRD was the independent variable. Average MU between 2000 and 2012 (a linear scale ranging from 1 to 7) was the dependent variable. Age, gender, SES indicators, and substance use by friends and parents were covariates. In the first step, we estimated a model in the pooled sample without the interaction term (Model 1). In the next step, the gender by PRD interaction term was added to the model (Model 2). As the final step, we estimated regression models specific to each gender (Model 3 and Model 4).

### Power Calculation

We used differences in means for power calculation. Our power analysis revealed that an overall sample size of 546, composed of 273 males and 273 females would give us ample statistical power (>0.80) to detect a gender differences in PRD. Appendix 1 in supplementary material shows a summary of the information that we used for power calculation in this study.

### Missing Data Analysis

We used list wise deletion to handle the missing data. Our missing data analysis revealed that age, gender, SES, and baseline PRD were not significantly different between individuals who entered and those who were excluded from analysis.

## Results

### Descriptive Statistics

Table 1 shows the descriptive statistics in the pooled sample and the gender stratified samples. Males reported more baseline PRD than females (*p* < 0.05). Males also reported more MU over the follow up years compared to females (*p* < 0.05). Parental and peer substance use were not statistically different between male and female participants (*p* > 0.05).

**Table 1:** Descriptive statistics in overall sample and by gender.

	**All**		**Males**		**Females**	
	***n***	***%***	***n***	***%***	***n***	***%***
**Gender**						
Female	317	53.28	–	–	–	–
Male	278	46.72	–	–	–	–
SES parents married (wave 1)^*^						
No	436	74.15	186	68.13	250	79.37
Yes	152	25.85	87	31.87	65	20.63
**SES parental employment—two parents working (wave 1)**						
No	295	51.94	133	49.44	162	54.18
Yes	273	48.06	136	50.56	137	45.82
**Substance use by parents (wave 1)**						
No	487	82.54	233	85.04	254	80.38
Yes	103	17.46	41	14.96	62	19.62
**Substance use by friends (wave 1)**						
No	173	29.98	74	27.51	99	32.14
Yes	404	70.02	195	72.49	209	67.86
**MU (wave 2)**						
No	268	47.52	131	49.81	137	45.51
Yes	296	52.48	132	50.19	164	54.49
	***Mean***	***SD***	***Mean***	***SD***	***Mean***	***SD***
Age (Year) (Wave 1)	14.84	0.65	14.90	0.66	14.80	0.63
PRD (Wave 5)^*^	0.79	0.85	0.85	0.84	0.75	0.85
MU Average (Waves 6–12)^*^	3.96	3.38	4.76	3.82	3.25	2.76

*Source: FAS, flint adolescent study (1994–2012); PRD, perceived racial discrimination; SES, socioeconomic status; MU, marijuana use*;

* *p < 0.05 for comparison of males and females*.

### Bivariate Correlations

Table 2 shows the results of bivariate correlations in the pooled sample and the gender stratified sample. In the pooled sample, the positive correlation between baseline MU and average MU over time was stronger for males than females. In the stratified sample, we found a positive correlation between baseline PRD and average MU over time in males but not females.

**Table 2:** Bivariate correlations in the overall sample and by gender.

	**1**	**2**	**3**	**4**	**5**	**6**	**7**	**8**	**9**
**ALL**									
1 Gender (Male)	1	0.08^#^	0.13^**^	0.05	−0.06	0.05	0.06	−0.04	0.22^**^
2 Age (Year) (Wave 1)		1	−0.11^**^	−0.10^*^	0.09^*^	0.08^*^	−0.06	0.10^*^	−0.03
3 SES Marital status (Wave 1)			1	0.15^**^	−0.10^*^	−0.01	0.05	−0.16^**^	−0.05
4 SES Two parents working (Wave 1)				1	−0.07^#^	−0.01	−0.03	−0.05	−0.03
5 Substance use by parents (Wave 1)					1	0.21^**^	0.06	0.23^**^	0.22^**^
6 Substance use by friends (Wave 1)						1	0.14^**^	0.32^**^	0.18^**^
7 PRD (Wave 5)							1	0.03	0.08
8 MU (Wave 2)								1	0.23^**^
9 MU Average (Waves 6–12)									1
**MALES/FEMALES**									
1 Gender (Male)	−	−	−	−	−	−	−	−	-
2 Age (Year) (Wave 1)	−	1	−0.16^**^	−0.10^#^	0.16^**^	0.16^**^	−0.11	0.11^#^	−0.03
3 SES Marital status (Wave 1)	−	−0.08	1	0.14^*^	−0.09	0.03	0.14^#^	−0.15^*^	−0.09
4 SES Two parents working (Wave 1)	−	−0.10^#^	0.14^*^	1	−0.06	−0.13^*^	0.00	−0.04	−0.08
5 Substance use by parents (Wave 1)	−	0.04	−0.10^#^	−0.08	1	0.23^**^	0.14	0.20^**^	0.28^**^
6 Substance use by friends (Wave 1)	−	0.01	−0.05	0.09	0.21^**^	1	0.14	0.31^**^	0.22^**^
7 PRD (Wave 5)	−	−0.03	−0.03	−0.05	0.02	0.11^#^	1	−0.01	0.18^*^
8 MU (Wave 2)	−	0.10	−0.17^**^	−0.05	0.26^**^	0.34^**^	0.05	1	0.31^**^
9 MU Average (Waves 6–12)	−	−0.07	−0.06	0.01	0.21^**^	0.12^*^	−0.04	0.17^**^	1

*Source: FAS, flint adolescent study (1994–2012), PRD, perceived racial discrimination; MU, marijuana use; SES, socioeconomic status; Males Upper Diagonal; Females Lower Diagonal*,

* *p < 0.05*,

** *p < 0.01*,

# *p < 0.1*.

### Regression Models in the Pooled Sample

Table 3 summarizes the results of two linear regression models in the pooled sample. In both linear regression models, baseline PRD was the independent variable, average MU over time was the dependent variable, and age, gender, and SES were covariates. Based on Model 1, PRD was not associated with MU in the pooled sample. Based on Model 2, a significant interaction was found between the effects of gender and baseline PRD on average MU between 2000 and 2012 in the pooled sample, suggesting stronger effect of baseline PRD on average MU between 2000 and 2012 for males than females.

**Table 3:** Summary of linear regressions in the total sample.

	**Main effects**			**Main effects + Interaction**		
	***b***	**95% CI**	***p***	***b***	**95% CI**	***p***
Gender (Male)	1.66	0.99–2.33	0.000	0.99	0.08–1.90	0.032
Age (Year) (Wave 1)	−0.33	−0.87–0.20	0.221	−0.31	−0.84–0.22	0.255
SES marital status (Wave 1)	−0.02	−0.77–0.74	0.968	−0.09	−0.84–0.66	0.809
SES parental employment—two parents working (Wave 1)	−0.48	−1.15–0.18	0.151	−0.50	−1.16–0.16	0.136
Substance use by parents (Wave 1)	1.27	0.35–2.20	0.007	1.21	0.29–2.12	0.010
Substance use by friends (Wave 1)	0.32	−0.44–1.07	0.413	0.31	−0.45–1.06	0.424
MU (Wave 2)	1.22	0.49–1.95	0.001	1.27	0.55–2.00	0.001
PRD (Wave 5)	0.17	−0.22–0.56	0.394	−0.17	−0.68–0.33	0.494
PRD (Wave 5) × Gender (Male)	−	–	–	0.85	0.06–1.63	0.034
Constant	7.27	−0.69–15.22	0.073	7.18	−0.73–15.10	0.075

*Source: FAS, flint adolescent study (1994–2012); PRD, perceived racial discrimination; MU, marijuana use; SES, socioeconomic status*.

### Regression Models by Gender

Table 4 summarizes two linear regression models, one for males and one for females. In both models, baseline PRD was the independent variable, average MU between 2000 and 2012 was the dependent variable, and age and SES were the covariates. Based on Model 3, baseline PRD was not associated with average MU between 2000 and 2012 in Black females. Based on Model 4, we found an association between baseline PRD and average MU between 2000 and 2012 in Black males, which was significant net of all covariates.

**Table 4:** Summary of linear regressions in males and females.

	**Males**			**Females**		
	***b***	**95% CI**	***p***	***b***	**95% CI**	***p***
Age (Year) (Wave 1)	−0.32	−1.23–0.58	0.484	−0.42	−1.08–0.23	0.203
SES marital status (Wave 1)	−0.10	−1.31–1.11	0.869	−0.37	−1.33–0.60	0.454
SES parental employment [Two Parents Working (Wave 1)]	−0.68	−1.82–0.45	0.235	−0.23	−1.03–0.58	0.575
Substance use by parents (Wave 1)	1.39	−0.28–3.07	0.102	1.09	0.03–2.15	0.043
Substance use by friends (Wave 1)	0.77	−0.61–2.15	0.269	−0.10	−1.00–0.80	0.827
MU (Wave 2)	2.11	0.88–3.34	0.001	0.63	−0.25–1.51	0.158
PRD (Wave 5)	0.68	0.00–1.35	0.050	−0.11	−0.57–0.35	0.633
Constant	7.66	−5.77–21.10	0.261	9.38	−0.36–19.11	0.059

*Source: FAS, flint adolescent study (1994–2012), PRD, perceived racial discrimination; MU, marijuana use; SES, socioeconomic status*.

## Discussion

We found gender differences in the prospective, longitudinal effect of PRD during adolescence (in 1999) on average MU between 2000 and 2012 during emerging adulthood and adulthood. Specifically, high PRD during adolescence was predictive of subsequent MU among Black males, but not Black females. To guide our understanding of why the influence of PRD on MU varies by gender, we situated our results within several theoretical frameworks and empirical research on gender differences in coping, prejudice, and stereotypes. We also propose a role for racial identity and masculine ideologies in explaining gender differences with regards to PRD-related consequences.

Our study is not the first to document gender variations in the consequences of PRD. Researchers have found that the effects of PRD on several undesired mental health outcomes from psychological distress and depression to suicide and substance use differ by sex (31, 35, 36, 38). In addition to gender, socioeconomic status (23, 57, 58), racial identity (59), race socialization and self-esteem (60) also alter sensitivity to PRD, which is more commonly reported by high SES Blacks, particularly males who are in proximity of Whites (61–63). Substance use is shown to be used as a way for coping with PRD (64). Substance use is one of many consequences associated with PRD for Black youth (65–67). Nevertheless, the underlying mechanism behind males' higher vulnerability to the effects of PRD on substance use is unknown. We know that White men may specifically target Black men, as they have higher implicit bias than White women do against Blacks (61). These findings are in line with the subordinate male target hypothesis (SMTH) which claims that “the discrimination experienced by the men of subordinate groups—primarily at the hands of men of dominant groups—is greater than that experienced by the women of the same subordinate groups” [(68), page 646].

Our findings support previous researchers who found that males are more sensitive to the effects of PRD on substance use and distress than females (31, 36). Being male (69) and having higher SES (62, 69) augments the likelihood of PRD exposure for Black Americans. In a study by Assari et al. (74), an increase in PRD during adolescence predicted an increase in depressive symptoms among male but not females. In another study among Black youth, neighborhood stress was predictive of major depressive disorder (MDD) in males, but not females (70). Likewise, stressful life events at baseline had a stronger predicted role for future MDD risk in Black males than females (36). These results suggest that stressful life events may have larger effects on psychopathology and substance use in males than females (70, 74). These finding are not specific to Blacks (71) and has been documented among Arab Americans (36). Overall our study adds to the literature that males appears to have higher vulnerability compared to females to the mental health effects of PRD. These differences may be in part because males and females have different levels or access to protective factors, such as religiosity and social support. Religion and social support may also have differential effects for males and females (72, 73).

While scholars have documented gender differences in associations between PRD and substance use (74), we are among the first to observe that the long term influence of PRD on MU also varies by gender. Our results, consistent with other researchers, suggest that Black males have long term increased vulnerability to substance use in the context of racism-related experiences (43, 75).

Our results also suggest that Black males and females may respond differently to PRD-related stress. In a longitudinal study of adolescent PRD on adult health behavior, Black males showed a tendency to turn to substance use while females had a higher tendency to reduce exercise and physical activity and increase binge eating within the context of PRD (31, 39). To this end, gender differences in coping with stress may explain the observed gender differences in the effect of PRD on MU. Black females have a higher tendency to use avoidant coping mechanisms in response to race related stress compared to Black males (76). Black males, in contrast, have a higher tendency to use effortful coping styles (77), which, in turn, can increase risk of substance misuse. While PRD is a social stressor, it can promote psychological and behavioral risks, such as hyper-vigilance, social isolation, negative emotions, and increased risk of unhealthy behaviors (5, 24, 78). In addition to the emotional toll of PRD (23), PRD may also shorten telomere length (79), and alter cortisol concentration (80).

In contrast to males who may have a tendency to turn to substance use, PRD for Black females, has stronger negative effects on obesity, diet, and exercise (44, 45, 81). In a study, PRD had a stronger effect on eating disorders for Black females than Black males (39). This pattern is not specific to PRD, but also for other types of environmental stressors, such as neighborhood stress. Several researchers have documented stronger effects from fear of neighborhood on obesity and body mass for Black females than Black males (81). These findings are consistent with hypothesis proposed by James Jackson on gender-specific response to stress among Blacks (82).

While PRD predicts substance use (83), this effect may depend on racial identity (5, 59, 84–86), racial attribution (26), and masculinity (87). That means, these social and psychological constructs may explain the gendered results in studies of PRD and mental health. Early PRD may also moderate the effect of later PRD on health outcomes (88). Masculine role norms, for instance, moderated the link between PRD and depressive symptoms in Black males (89). PRD and environmental stress is more strongly linked to psychopathology for males who have high vs. low masculine ideologies (87). Restricted emotionality and self-reliance, which follow masculinity, increases the risk for distress and depression (89). High racial identity (e.g., centrality) may increase the effects of PRD on depression (90).

Structural racism at multiple levels including media may be partially responsible for higher exposure and vulnerability of Black males to PRD. Black males are hyper-stereotyped as aggressive, intimidating, and violent, and as a result are more likely to be punished and expelled from school and searched and, at times, brutalized by law enforcement than their white counterparts (91, 92). Black males, in turn, may develop a heightened vulnerability to PRD over the life course (62). As systemic racism related exposures (e.g., police brutality, mass incarceration, stop and frisk) disproportionately target Black males (93), the effects of PRD on stress-related coping behaviors like MU may be magnified among males. At the same time, Black males receive more parental messages regarding racism and discrimination compared with the Black females (94, 95) which in turn may increase their vigilance for discriminatory cues.

Stereotypes about Blacks that exist in the media and other public domains are not shaped merely by race, but the intersection of race and gender. For instance, media has a higher tendency to portray Black males as aggressive and anti-intellectual (86, 96–98) than Black females (or any other group). In the US, Black males have been stereotyped as “endangered, aggressive, angry, superhuman, subhuman, lazy, hyperactive, jailed, and paroled, on probation, lost, loveless, incorrigible, or just simply self- destructive” [(99), p. 185; (100)]. As a result, the distorted understanding and attitudes of Black males can have real-world consequences, including increased and persistent exposure to institutional and interpersonal racial discrimination. At the individual-level, exposure to racial stereotypes have been linked to negative attitudes and beliefs about one's race. In other words, PRD experienced by Black males is not just “perceived” but real with health consequences.

Among Blacks, males report higher PRD compared to females (86, 90, 97, 101, 102). In a recent study, darker skin color, a construct closely associated with PRD among Blacks, was predictive of PRD in male, but not female Black youth (70). Ifatunji and Harnois (47) proposed two hypotheses to explain the existing gender gap in PRD. Based on subordinate male hypothesis, across race and ethnic groups, males are considered as the main threat. As a result, most between-group conflicts happen between males across racial/ethnic groups. In this view, Black males experience more discrimination in comparison to Black females. Based on the race-gender intersectionality hypothesis, gender causes bias in measurement of PRD. Ifatunji and Hamois (47) found evidence suggesting that gender differences in experiences of major life discrimination tend to be due to measurement bias, however, gender differences in experience of everyday discrimination (as we measured in our study) are better justified by subordinate male hypothesis (47).

### Implications

These findings have implications for research, clinical practice, as well as public policy. Drug use and misuse prevention programs for Black youth may be more effective if they address race-related discrimination. In particular, culturally tailoring the treatment of substance use disorders should entail assessing and addressing PRD when treating Black individuals, and especially Black males. Clinicians may want to screen for or discuss PRD when providing services for substance abuse prevention, diagnosis and treatment of Black male youth. Racial discrimination currently occurs in schools, criminal justice, neighborhoods, labor markets, and almost all other institutions. Civil society and governments must do more to eliminate racial discrimination for all groups, including Blacks (103).

Future research that examines how multiple facets of racial discrimination, structural factors, and individual factors shape how Black males cope with PRD. In addition to neighborhood context and economic factors, research should include individual factors, such as identity, attribution, and coping strategies/styles. Emerging research is beginning to document that high SES may be associated with worse mental health among Black males (104–107). Further research is warranted to uncover social, psychological, and behavioral mechanisms behind the heightened effects of PRD in males. Two potential suspects are hegemonic masculinity (89) and John Henryism (108) which may increase vulnerability of Black males to PRD. Future research should test whether differential use of avoidant and confronting coping explain any of gender differences in vulnerability to PRD. For example, Black males are more frequently exposed to neighborhood crime and violence (76). Black males also do not similarly benefit from the same protective factors as Black females, such as social support (109–112) and religious involvement (113). It is, therefore, important to understand the risk and protective factors that are unique to Black males within the context of PRD. Gender differences in psychological, social, and behavioral risk factors linked to PRD may be crucial to explaining why Black males are more vulnerable to some of psychological consequences of PRD (e.g., substance use) than Black females. High vulnerability to PRD in high SES Black males may also explain poor mental health of high SES Black males (104–106). Additional research is needed to develop an interdisciplinary understanding of how gender, context, and culture interact to shape the emotional and behavioral response to PRD among Black males and females (86). MU may become a more common way to cope with stress and PRD in near future as more states legalize recreational MU.

### Limitations

This study has several limitations. First, few items were used to measure MU. Future research should use structured interviews or comprehensive measures to collect more detailed data on MU. Second, we did not study other substances, such as tobacco, alcohol, or other drugs. This is especially important because our data were collected before marijuana medicalization and legalization policies were enacted in many states in the U.S. It would be useful to see if PRD is an antecedent of substance use more generally to determine if alcohol and other drugs are used as a coping mechanism for PRD (i.e., self-medication). We also did not measure psychiatric disorders, such as depression as well as history of depressant treatment. Third, the study did not measure other sources of discrimination, such as gender discrimination. Fourth, the data were old, but the experiences of PRD may not be time bound and although MU may have become more socially (and legally) acceptable, its use as a coping strategy for discrimination remains a significant social policy concern. In other words, we have no reason to believe that the fundamental finding that substances are used at higher rates among Black males who report racial discrimination would be different today than when the data in this study were collected. In fact, our findings are consistent with findings from more recent data but add important new information to the literature. Fifth, although MU was measured multiple times, PRD was only measured at baseline. The fact that we found early PRD to have persistent effects many years later, however, is a unique contribution of this study. Future research may explore the cumulative effect of PRD over multiple time points in the life course on marijuana and substance use more generally. Sixth, we did not consider racial identity, attribution, and coping. Racial identity, attribution style, and coping could all alter the effects of PRD on a wide range of health outcomes. Finally, our analysis did not use the most effective approach for understanding longitudinal data, but we used linear regression because our outcome of interest, MU, was the average of MU over time vs. examining MU at various time points. Our results suggest that future research should examine trajectories of MU using techniques, such as generalized estimating equations, time series, growth curve modeling, or mixed effects regression to understand temporal covariations between PRD and MU. Despite these limitations, this study extends the existing literature on the intersections of race, gender, PRD, and MU, as it is one of very few studies that has explored gender differences in the effects of PRD on MU over a long period of time. A significant contribution of this study is that we examined these relationships among more than five hundred Black youth over 18 years.

### Conclusion

Black males might be more vulnerable than Black females to the long-term effects of PRD on MU. Further research is needed to identify social, psychological, and behavioral mechanisms that may explain why Black males are particularly vulnerable to the mental health consequences of PRD. Clinicians may consider PRD as a more salient determinant of poor behavioral and mental health, particularly in Black males. Although we found that males are more prone to the effects of PRD on MU, discrimination should be prevented for all groups regardless of race or gender. No population subgroup should be marginalized, stigmatized, or stereotype, for their skin color, or any other characteristics. Strengthening existing anti-discrimination laws may improve prevention of MU in Black males, however, such policies should be multi-level.

## Ethics Statement

The FAS protocol was approved by the University of Michigan (UM) Institutional Review Board (IRB). All participants signed consent or assent forms before each interview, depending their age at each observation. Working with the schools, we used passive consent for parents for the initial data collection in 1994. Participants received financial compensation for participating in the study. All the sensitive data were collected after the interview using paper and pencil questionnaires. Respondents were informed that all the collected data would be kept confidential and we obtained a certificate of confidentiality from the National Institutes of Health.

## Author Contributions

The original idea of this analysis was developed by SA. SA also analyzed the data. All authors collaborated on interpretation of the data, drafting the manuscript, and revising the paper. All authors confirmed the final version of the manuscript. MZ is the principle investigator of the FAS.

### Conflict of Interest Statement

The authors declare that the research was conducted in the absence of any commercial or financial relationships that could be construed as a potential conflict of interest. The reviewer BA and handling Editor declared their shared affiliation at the time of review.
