# Supplementary material for: Perceived Racial Discrimination and Marijuana Use a Decade Later; Gender Differences Among Black Youth
Source: Front Pediatr. 2019 Mar 22;7:78. doi: 10.3389/fped.2019.00078 (PMC6438901; doi:10.3389/fped.2019.00078)
Supplement: Supplementary file 1 [file Data_Sheet_1.docx]

**Appendix 1**

**Sample size calculation**

### Sample Size for Comparing Two Means

| Confidence Interval (2-sided) | | 95% |  | | |  |
| --- | --- | --- | --- | --- | --- | --- |
| Statistical Power | | 80% |  | | |  |
| Ratio of Sample Size (Males/Females) | | 1.00 |  | | |  |
|  | | | | | |  |
|  | **Males** |  | **Females** |  | **Difference*** |  |
| Mean Perceived Discrimination | 0.85 |  | 0.70 |  | 0.15 |  |
| Standard Deviation Perceived Discrimination | 0.65 |  | 0.60 |  | | |
| Variance | 0.42 |  | 0.36 |  | | |
|  | | | | | |  |
| Sample size of males | | 273 |  |  |  |  |
| Sample size of females | | 273 |  |  |  |  |
| Total sample size | | 546 |  |  |  |  |

In the introduction of the research, the effect of PRD on men is emphasized. However, it is important to point out the effects of PRD on women from a holistic point of view.

It should be noted how the sample size of the study is calculated in the material method section.

In the study, why 4 schools were chosen should be explained in more detail.

comprehensive recommendations should be made for PRD prevention.
